# Embryonic lethality and defective male germ cell development in mice lacking UTF1

**DOI:** 10.1038/s41598-017-17482-z

**Published:** 2017-12-08

**Authors:** Seth D. Kasowitz, Mengcheng Luo, Jun Ma, N. Adrian Leu, P. Jeremy Wang

**Affiliations:** Department of Biomedical Sciences, University of Pennsylvania School of Veterinary Medicine, Philadelphia, PA 19104 USA

## Abstract

The germ cell lineage is specified early in embryogenesis and undergoes complex developmental programs to generate gametes. Here, we conducted genetic studies to investigate the role of *Utf1* (Undifferentiated embryonic cell transcription factor 1) in mouse germ cell development. *Utf1* is expressed in pluripotent embryonic stem (ES) cells and regulates ES cell differentiation. In a proteomics screen, we identified UTF1 among 38 proteins including DNMT3L and DND1 that associate with chromatin in embryonic testes. We find that UTF1 is expressed in embryonic and newborn gonocytes and in a subset of early spermatogonia. Ubiquitous inactivation of *Utf1* causes embryonic lethality in mice with a hybrid genetic background. Male mice with a germline-specific deletion of *Utf1* resulting from *Prdm1*-Cre mediated recombination are born with significantly fewer gonocytes and exhibit defective spermatogenesis and reduced sperm count as young adults. These defects are ameliorated in older animals. These results demonstrate that UTF1 is required for embryonic development and regulates male germ cell development.

## Introduction

In sexually reproducing organisms, germ cells pass genetic information to the next generation. The germ cell lineage arises in early embryogenesis. Toward the end of gastrulation, murine primordial germ cells (PGCs) arise from the epiblast and, as a small cluster of cells, adopt a germ cell fate outside the embryo proper^1,2^. Subsequently, PGCs proliferate and migrate to the bipotential genital ridges, which develop into either testes or ovaries. Following sex determination at embryonic day 12.5 (E12.5), male germ cells are enclosed in seminiferous cords to become gonocytes. Gonocytes continue to proliferate and enter mitotic arrest at about E16^3^. Gonocytes undergo genome-wide epigenetic reprogramming and only resume proliferation shortly after birth^3^. In contrast, female germ cells enter meiosis, progress through early stages of meiosis I, and become arrested at the dictyate stage of meiosis I at birth. The number of oocytes is finite in females at birth. In contrast, males produce sperm continuously throughout adult life due to the presence of spermatogonial stem cells in the testis^3,4^. Spermatogonial stem cells, a subset of spermatogonia, are capable of self-renewal and differentiation. At puberty, spermatogonia enter meiosis to become spermatocytes, which give rise to post-meiotic haploid spermatids and, finally, to mature spermatozoa.

Embryonic stem (ES) cells are derived from the inner cell mass of the blastocyst. ES cells are pluripotent and capable of self-renewal and differentiation into different cell types. Undifferentiated embryonic cell transcription factor 1 (*Utf1*) is expressed in pluripotent ES cells and embryonal carcinoma cells, but is rapidly downregulated upon differentiation^5^. UTF1 is a transcription repressor protein that associates with chromatin in mouse ES cells in a dynamic manner similar to core histone proteins^6^. UTF1 prevents ES cell chromatin decondensation^7^. *Utf1* is a transcriptional target of OCT4 and SOX2^8,9^. Mechanistically, UTF1 modulates the epigenetic state of bivalent genes and prevents aberrant gene expression through mRNA decapping in ES cells^7,10^. These UTF1 functions are critical for ES cell pluripotency and differentiation. Indeed, knockdown or inactivation of *Utf1* causes a delay in ES cell differentiation^6,10^.

In mouse embryos, *Utf1* is expressed in the inner cell mass, the primitive ectoderm and the extra-embryonic tissue, but not in the mesoderm^5^. Downregulation of *Utf1* expression coincides with differentiation of the primitive ectoderm into mesoderm. *Utf1* is not expressed in most adult tissues except in testis and ovary. In rats, *Utf1* expression is restricted to a subpopulation of early spermatogonia in the adult testis^11^. In humans, *Utf1* is also expressed in spermatogonia^12^. The phenotype of *Utf1*-null mouse mutants depends on the strain background. In mice of C57BL/6J strain background, inactivation of *Utf1* causes embryonic developmental delay, resulting in lethality within 2 days after birth, whereas *Utf1*-null mice on a C57BL/6J × ICR mixed genetic background are viable and fertile^13^.

Here we report a role of UTF1 in embryogenesis and male germ cell development. In a proteomics screen, we identified UTF1 as one of 38 chromatin-associated proteins enriched in developing male embryonic germ cells. We find that UTF1 is present in the nucleus of embryonic male germ cells. Inactivation of *Utf1* results in embryonic lethality on the C57BL/6 × 129 hybrid genetic background. Furthermore, inactivation of *Utf1* leads to a reduction in the number of gonocytes at birth and defective spermatogenesis in adult males.

## Results

### UTF1 is a chromatin-associated protein in male embryonic germ cells

**Figure 1:**
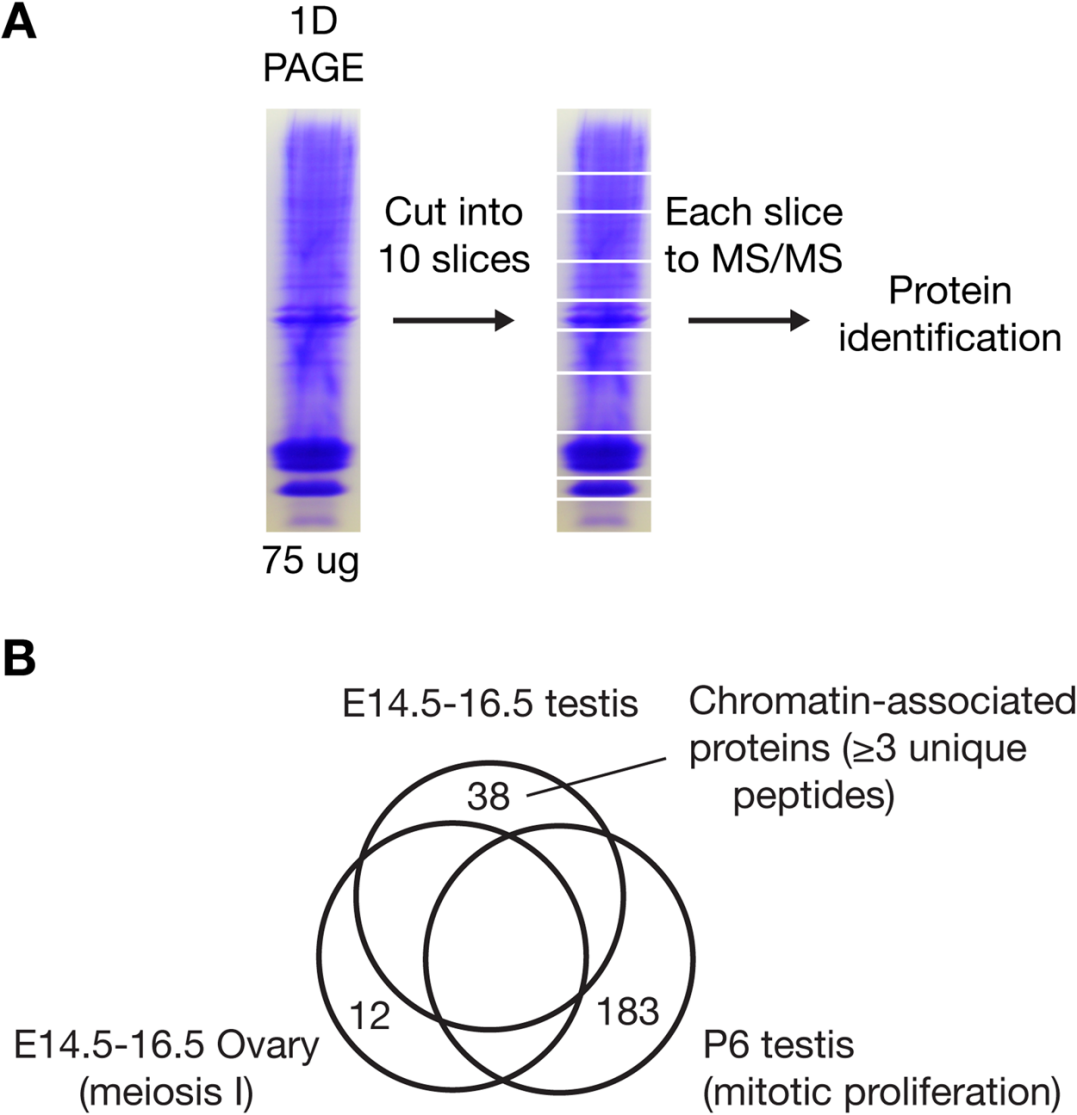
Proteomic identification of chromatin-associated proteins from E14.5-E16.5 testes. (**A**) Experimental approach. Identification of chromatin-associated proteins from E14.5-16.5 embryonic mouse testes was used here. (**B**) Systematic identification of chromatin-associated proteins enriched in E14.5-E16.5 testes by subtractive analysis of three proteomic datasets from E14.5-E16.5 testes (771 proteins, each with at least three unique peptides), E14.5-E16.5 ovaries (534 proteins), and postnatal day 6 (P6) testes (1011 proteins). The number of proteins with at least 3 unique peptides in one dataset but none in the other two datasets are shown.

We designed a proteomic screen to identify proteins that are associated with the chromatin of embryonic gonocytes in the developing mouse testis. Adapting our previously described methodology^14^, we performed a mass spectrometry analysis of chromatin-associated proteins from embryonic (E14.5-16.5) testes (Fig. 1A) and ovaries, respectively. At this stage of development, male germ cells (called gonocytes) still proliferate but undergo genome-wide epigenetic reprogramming, whereas female germ cells enter the prophase of meiosis I^3^^,^^15^. In the post-natal day 6 (P6) testis, germ cells (now called spermatogonia) proliferate by mitosis. To identify proteins specific to or enriched in male embryonic germ cells, we subtracted all proteins also found in E14.5-16.5 ovary and/or P6 testis. With this approach, we excluded any chromatin-associated proteins that are common to meiotic germ cells of the embryonic ovary and spermatogonia of the P6 testis and also eliminated proteins from somatic cell types of the testis, which are present in both embryonic and post-natal testes. Using a cutoff of at least three unique peptides, we identified 38 chromatin-associated proteins in embryonic male germ cells (Fig. 1B and Table 1).

**Table 1:** Chromatin-associated proteins identified in male embryonic germ cells.

Protein	Gene Accession	Protein Accession	No. Unique Peptides	Percent Coverage	Length (aa)	Official Full Name
UTF1	NM_009482	NP_033508	6	32%	340	Undifferentiated embryonic cell transcription factor 1
DND1	NM_173383	NP_775559	6	23%	353	DND microRNA-mediated repression inhibitor 1
DNMT3L	NM_001081695	NP_001075164	5	16%	422	DNA (cytosine-5)-methyltransferase 3-like
WDR43	NM_175639	NP_783570	5	11%	678	WD repeat domain 43
ZFP326	NM_018759	NP_061229	4	8%	581	Zinc finger protein 326
INTS7	NM_178632	NP_001293132	4	6%	967	Integrator complex subunit 7
UTP11L	NM_026031	NP_080307	4	15%	254	UTP11-like, U3 small nucleolar ribonucleoprotein
KDM3A	NM_001038695	NP_001033784	4	4%	1324	Lysine demethylase 3A
EXOSC10	NM_016699	NP_057908	4	9%	888	Exosome component 10
PHF10	NM_024250	NP_077212	4	12%	498	PHD finger protein 10
PPP2R5C	NM_001135001	NP_001128473	4	10%	510	Protein phosphatase 2, regulatory subunit B′, gamma
THOC2	NM_001033422	NP_001028594	3	2%	1595	THO complex 2
PPP2CB	NM_017374	NP_059070	3	13%	310	Protein phosphatase 2 (formerly 2A), catalytic subunit, beta isoform
WTAP	NM_175394	NP_780603	3	31%	152	Wilms tumour 1-associating protein
YLPM1	NM_178363	NP_848140	3	3%	2140	YLP motif containing 1
NOC2L	NM_021303	NP_067278	3	5%	751	NOC2 like nucleolar associated transcriptional repressor
SUPT6	NM_009297	NP_033323	3	3%	1727	Suppressor of Ty 6
CTNNB1	NM_001165902	NP_001159374	3	6%	782	Catenin (cadherin associated protein), beta 1
NFYC	NM_001048168	NP_001041633	3	10%	336	Nuclear transcription factor-Y gamma
KAT7	NM_177619	NP_808287	3	8%	523	K(lysine) acetyltransferase 7
LDB1	NM_001113408	NP_001106879	3	11%	412	LIM domain binding 1
ACTR2	NM_146243	NP_666355	3	13%	395	ARP2 actin-related protein 2
DDB1	NM_015735	NP_056550	3	4%	1141	Damage specific DNA binding protein 1
MRPS9	NM_023514	NP_076003	3	11%	391	Mitochondrial ribosomal protein S9
SCFD2	NM_001114660	NP_001108132	3	8%	685	Sec1 family domain containing 2
MLLT4	NM_010806	NP_034936	3	3%	1821	Myeloid/lymphoid or mixed-lineage leukemia; translocated to, 4
KDM2A	NM_001001984	NP_001001984	3	3%	1162	Lysine (K)-specific demethylase 2A
GFM2	NM_001146043	NP_001139515	3	6%	742	G elongation factor, mitochondrial 2
ATM	NM_007499	NP_031525	3	2%	3067	Ataxia telangiectasia mutated
ANAPC5	NM_001042491	NP_001035956	3	6%	728	Anaphase-promoting complex subunit 5
BZW2	NM_025840	NP_080116	3	12%	420	Basic leucine zipper and W2 domains 2
TCF20	NM_013836	NP_038864	3	3%	1966	Transcription factor 20
ACOT7	NM_133348	NP_579926	3	10%	380	Acyl-CoA thioesterase 7
CUL4B	NM_028288	NP_082564	3	5%	971	Cullin 4B
ARID4B	NM_194262	NP_919238	3	4%	1315	AT rich interactive domain 4B (RBP1-like)
SAP30BP	NM_020483	NP_065229	3	12%	309	SAP30-binding protein
NEK1	NM_175089	NP_001280568	3	4%	1204	NIMA (never in mitosis gene a)-related expressed kinase 1
CWC27	NM_026072	NP_080348	3	10%	470	CWC27 spliceosome-associated protein

Several proteins identified in our screen have a well-characterized role in germline development, including DND1 and DNMT3L (Table 1). DND microRNA-mediated repression inhibitor 1 (DND1) is essential for primordial germ cell migration and survival in zebrafish, and has a strong influence on differentiation and cell cycle control in the mammalian germ line^16,17^. A mutation in *Dnd1* causes germ cell loss and testicular germ cell tumors in mice^18^. DNA methyltransferase 3-like (DNMT3L) establishes maternal genomic imprints and maintains silencing of LINE1/IAP retrotransposons in the male germ line^19–21^. Deletion of *Dnmt3l* causes loss of embryos in knockout females and sterility in males. The identification of known proteins in this proteomics screen confirmed the validity of our approach and suggests that other proteins identified may also play an important role in the development of male embryonic germ cells.

**Figure 2:**
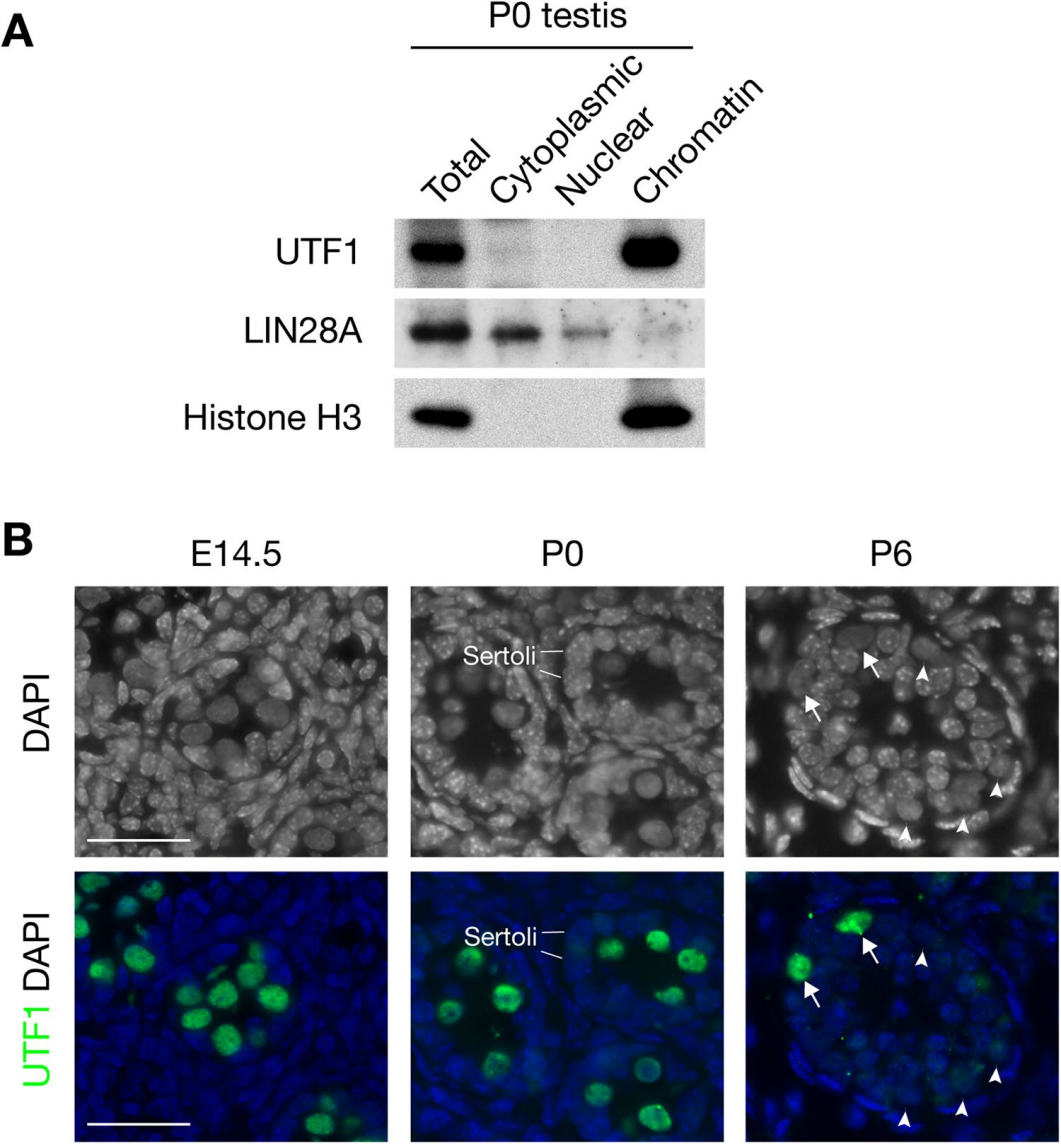
UTF1 is associated with chromatin in early male germ cells. (**A**) Western blot analysis of UTF1 in total and subcellular fractions of neonatal (P0) testis. LIN28A serves as a cytoplasmic protein control^35^. Histone H3 serves as a chromatin protein control. Images were cropped to show the relevant bands. The full-length blots are presented in Supplementary Figure S1. (**B**) Immunolocalization of UTF1 in sections from wild-type E14.5, neonatal (P0), and postnatal day 6 (P6) testes. DAPI staining of DNA shows the nuclear appearance of germ cells and supporting cells. Gonocytes (E14.5 and P0) and early spermatogonia (P6) lack heterochromatin and exhibit a uniform dark appearance, whereas somatic cells such as Sertoli cells are rich in heterochromatin visualized as punctate bright dots. In P6 testes, UTF1-positive and UTF1-negative spermatogonia are indicated by arrows and arrowheads respectively. Scale bars, 25 µm.

In our proteomics screen, UTF1 was the protein with the highest number of unique peptides (Table 1). Consistent with proteomic data, UTF1 was detected in the chromatin fraction of protein lysates from neonatal (P0) testes, but not in the cytoplasmic and soluble nuclear extracts (Fig. 2A). To assess the developmental and cell-type specific expression and intracellular localization of UTF1, we performed immunostaining of testis sections. UTF1 was present in the nuclei of all gonocytes in embryonic (E14.5) and P0 testes, but only in a subpopulation of spermatogonia in postnatal day 6 testis (Fig. 2B). UTF1 was not detected in spermatocytes or spermatids in postnatal testis. These results suggest that UTF1 may play a role in early male germ cell development.

### Ubiquitous *Utf1* deletion is embryonic lethal

**Figure 3:**
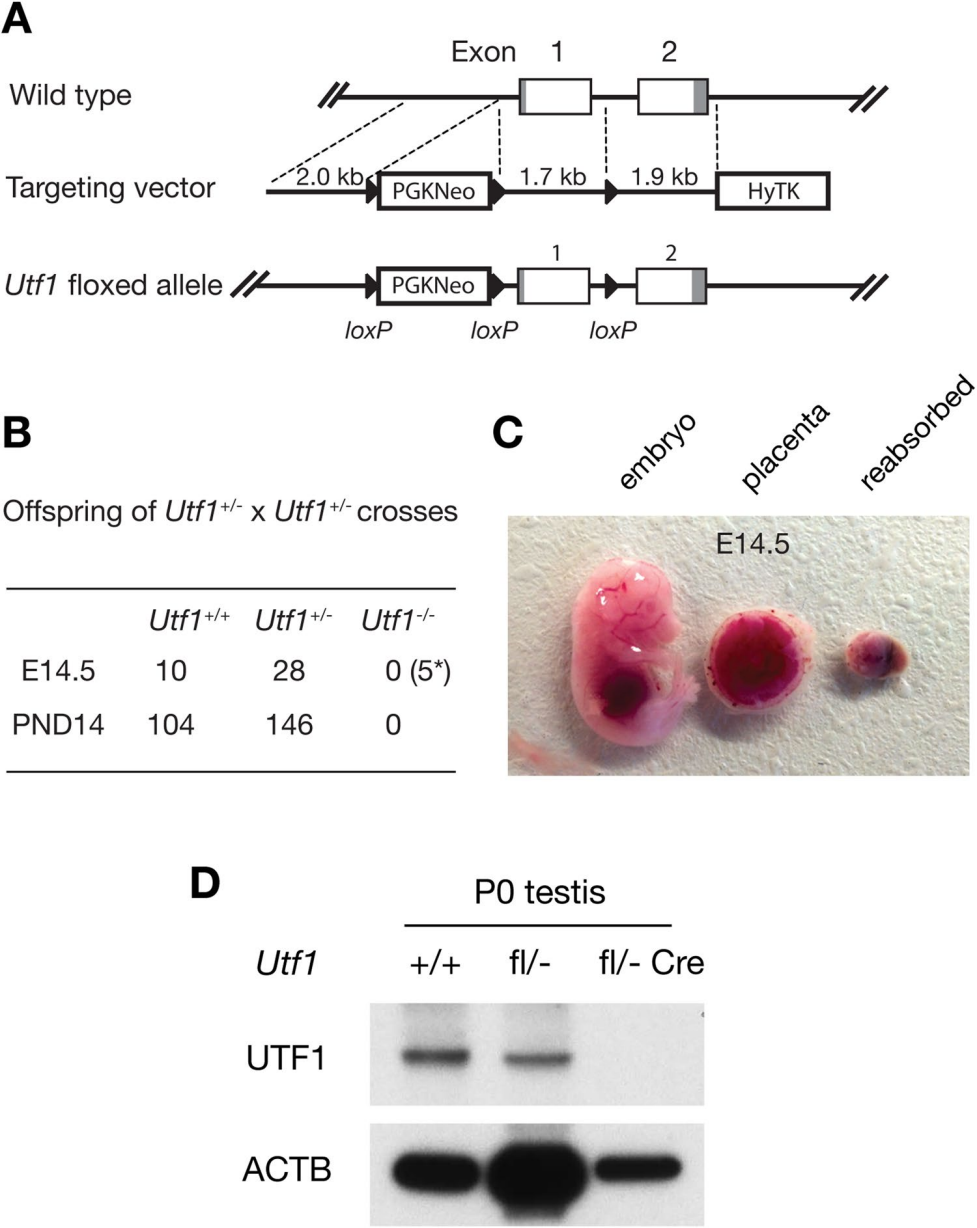
UTF1 is required for embryonic development. (**A**) Schematic diagram of the *Utf1* wild-type allele, targeting construct, and conditional knockout allele. Mouse *Utf1* gene maps to chromosome 7 and consists of two exons. (**B**) Embryos and pups from *Utf1* ^+/−^ × *Utf1* ^+/−^ matings. The number in the bracket labelled by * indicates the number of resorptions found. (**C**) E14.5 mouse embryos from *Utf1* ^+/−^ × *Utf1* ^+/−^ matings. Left and center: wild type embryo and placenta. Right: reabsorbed embryo of presumptive *Utf1* ^−/−^ genotype. (**D**) Absence of UTF1 protein in neonatal (P0) *Utf1* ^*fl*/−^ *Prdm1*-Cre testes. Note the reduction in UTF1 abundance in *Utf1* ^*fl*/−^ testes. ACTB serves as a loading control. The full-length blots are presented in Supplementary Figure S1.

To ascertain the tissue-specific requirement for *Utf1* in the gonad, we generated a conditional knockout (floxed) allele by homologous recombination in mouse ES cells. The *Utf1* gene is comprised of two exons located on mouse chromosome 7 and encodes a protein of 339 amino acids. In the conditional knockout allele, the first exon was flanked by *loxP* sites (*Utf1* ^*fl*^) (Fig. 3A), such that Cre recombinase-mediated deletion of exon 1 produces a null allele that lacks the potential promoter region and the coding sequence for the N-terminal 188 amino acids including the start codon. *Utf1* ^*fl/fl*^ mice were viable and fertile with no gross abnormalities.

From the intercross of *Utf1* ^*fl/fl*^ mice with *Actb-Cre* mice, in which Cre recombinase is expressed ubiquitously^22^, we obtained *Utf1* ^+/−^ mice. The *Utf1* ^+/−^ mice were on a mixed genetic background (FVB/N, C57BL/6, and 129). Interbreeding of *Utf1* heterozygous mice failed to yield any *Utf1* ^−/−^ offspring, and the genotype frequency of live-born pups was skewed towards the wild type (*Utf1* ^+/+^  = 104, *Utf1* ^+/−^ = 146) [χ^2^(1, *N* = 250) = 34.54, *p* < 4 × 10^−9^] (Fig. 3B). To determine the time of embryonic death, we performed timed heterozygote matings and isolated embryos from the uteri of *Utf1* ^+/−^ females at various embryonic stages. Homozygous *Utf1* ^−/−^ embryos were absent at E10.5 (N = 38, *Utf1* ^+/+^  = 10, *Utf1* ^+/−^ = 28, reabsorbed = 5) [χ^2^ = 4.29, *p* < 0.04] (Fig. 3B), however, residual placental tissues from reabsorbed embryos were detectable (Fig. 3C). This result demonstrates that *Utf1* is essential for early embryo viability.

### Conditional inactivation of *Utf1* causes a reduction in the number of gonocytes

**Figure 4:**
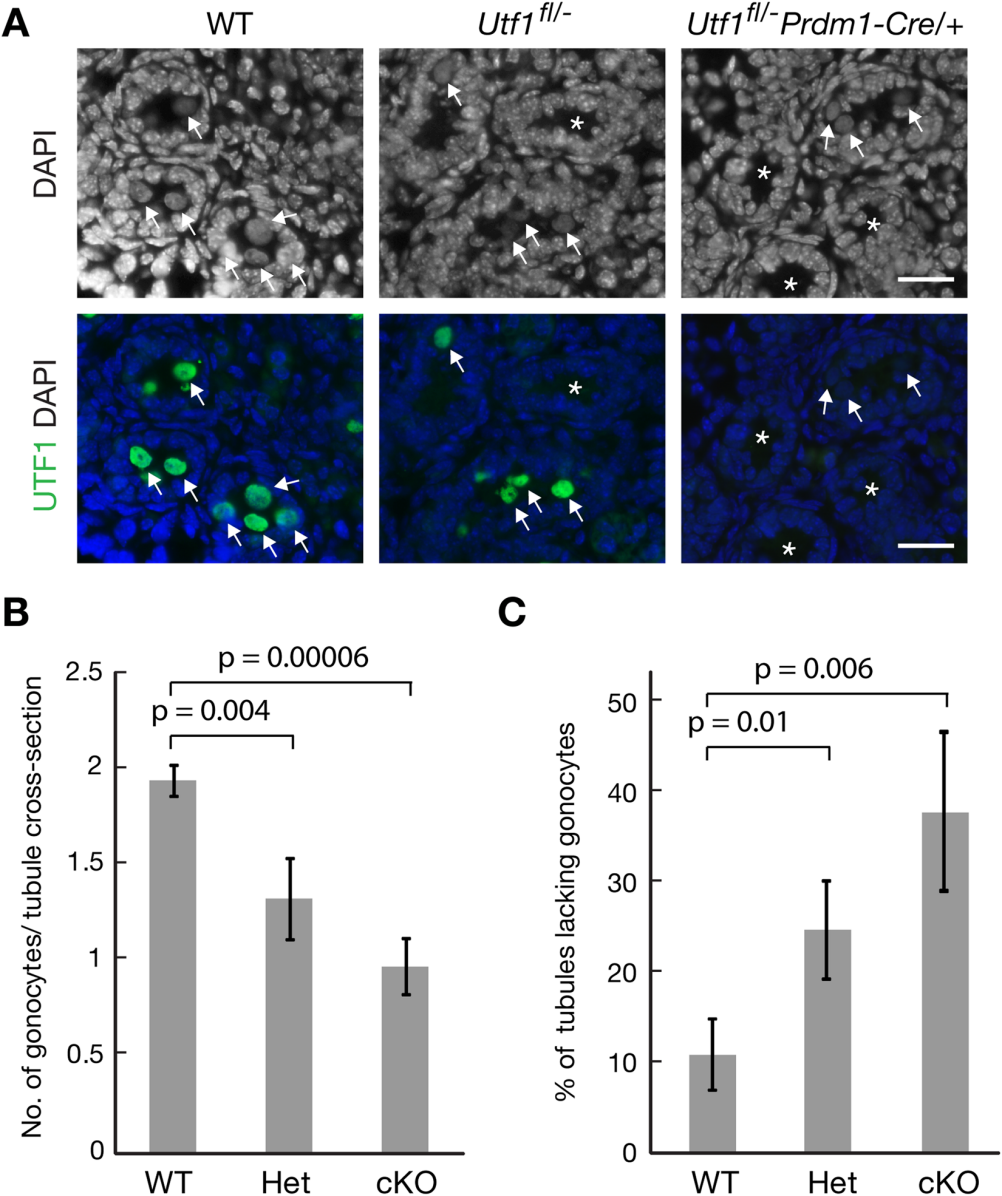
Reduced number of gonocytes in *Utf1* cKO neonatal testis. (**A**) Detection of gonocytes in seminiferous tubule cross-sections from newborn (P0) wild type (WT), *Utf1* ^*fl*/−^ (het), and *Utf1* ^*fl*/−^ *Prdm1-Cre/*+ (cKO) testes. Sections were immunostained with anti-UTF1 antibody and counterstained with DAPI. Gonocytes are indicated by arrows. Tubule cross-sections lacking gonocytes are designated by asterisks. Scale bars, 25 µm. (**B,C**) Quantitative analysis of gonocytes in seminiferous tubules from the testes of newborn WT (n = 4 animals), het (n = 5), and cKO (n = 6). For each animal, at least 150 tubule cross sections were counted. (**B**) Number of gonocytes per seminiferous tubule cross-section. **(C)** Percentage of tubules lacking gonocytes.

To elucidate the role of *Utf1* in germ cells, we inactivated the conditional allele using a mouse strain that expresses Cre recombinase under the control of the mouse *Prdm1* promoter (*Prdm1*-Cre). *Prdm1* expression is induced by BMP4 early in the post-implantation embryo^23^. PRDM1 is a marker of the founding population of primordial germ cells (PGCs), and *Prdm1*-Cre expression in PGCs begins at E6.25^23^. *Utf1* ^*fl/fl*^ mice were bred with *Prdm1-Cre* mice to produce *Utf1* ^*fl*/+^ *Prdm1-Cre* ^*tg*/+^ mice, which were then crossed with *Utf1* ^*fl/fl*^ females to produce *Utf1* ^*fl*/−^ *Prdm1-Cre* ^*tg*/+^ mice (referred to as *Utf1* cKO). The *Utf1* cKO mice were on a mixed genetic background (C57BL/6 × 129). Western blot analyses showed that UTF1 protein was absent in *Utf1* cKO testes and reduced in abundance in *Utf1* ^*fl*/−^ testes (Fig. 3D). Immunostaining of testis sections from newborn mice confirmed that UTF1 was present in the nucleus of both wild-type and *Utf1* ^*fl*/−^ gonocytes, but absent in *Utf1* cKO gonocytes (Fig. 4A), indicating that Cre-mediated deletion was complete at birth. Gonocytes were present in the testes of *Utf1* cKO neonate males after early embryonic deletion of *Utf1*. However, in both heterozygous and cKO testes, a subset of tubules were apparently devoid of gonocytes (Fig. 4A). Quantitative analysis revealed that *Utf1* ^*fl*/−^ and cKO testes contained significantly fewer gonocytes compared with wild-type testes, with a relatively larger reduction of gonocyte number in cKO testes (Fig. 4B). Consistent with this observation, the percentage of tubules lacking gonocytes was significantly higher in *Utf1* ^*fl*/−^ and *Utf1* cKO testes than in the wild type, again with a relatively larger increase in *Utf1* cKO testes (Fig. 4C).

### Conditional inactivation of *Utf1* causes spermatogenic defects in adult mice

**Figure 5:**
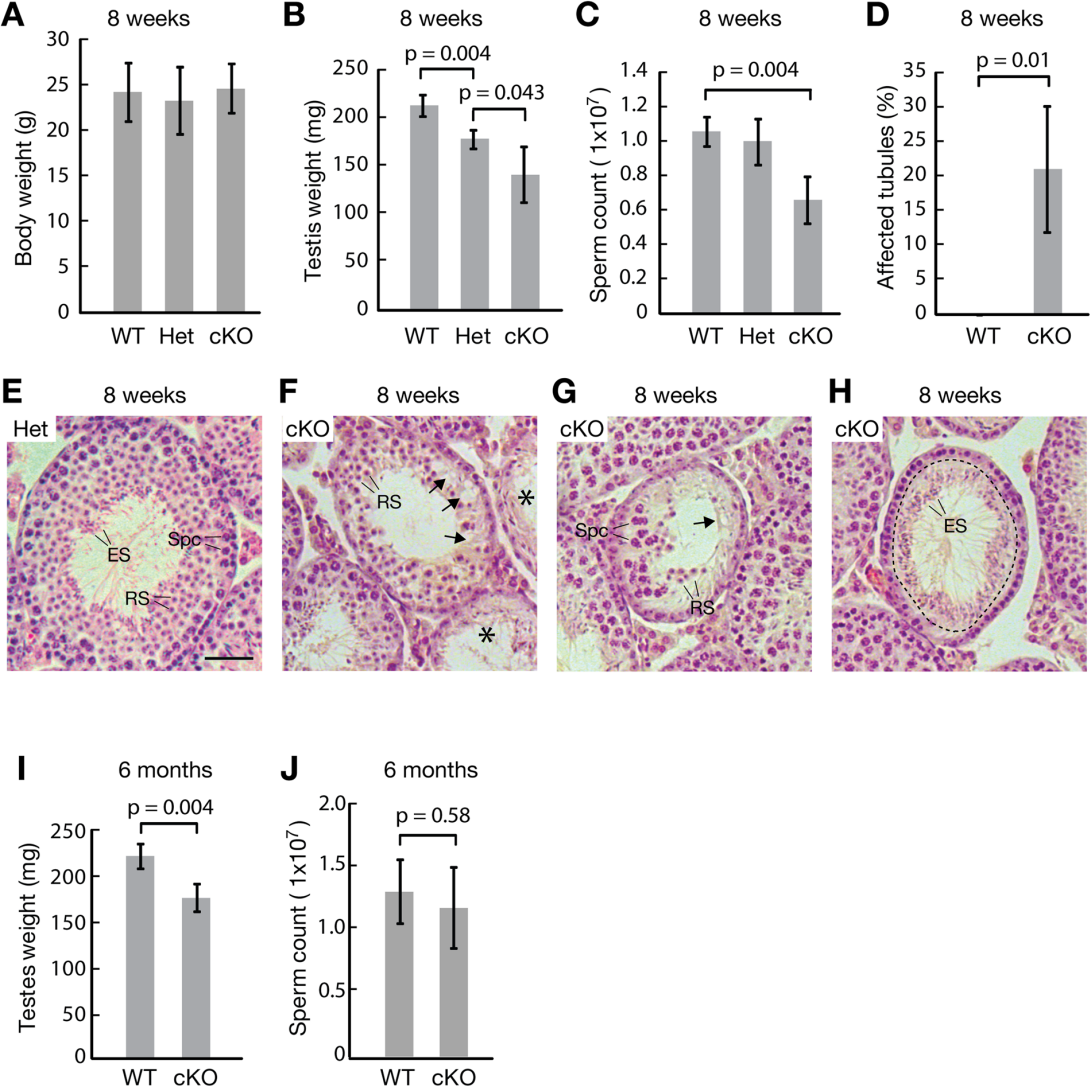
Impaired spermatogenesis in adult *Utf1* cKO males. **(A–D)** Phenotype of adult (8-week-old) wild-type (n = 4), heterozygous (n = 3), and *Utf1* ^*fl*/−^ *Prdm1-Cre* (cKO) males (n = 5). (**A**) Body weight. (**B**) Testis weight. (**C**) Sperm count. (**D**) Percentage of tubules with defective spermatogenesis. Only cross-sections were counted. Histological analysis of testes from 8-week-old *Utf1* ^f/−^ (**E**) and cKO (**F**–**H**) males. Arrows indicate areas of germ cell loss (**F**,**G**), asterisks tubules depleted of nearly all germ cells (**F**), and a dashed circle layers of missing germ cells (**H**). Abbreviations: Spc, spermatocytes; RS, round spermatids; ES, elongating spermatids. (**I,J**) Phenotype of 6-month-old wild-type (n = 3) and cKO (n = 4) males. **(I)** Testis weight. (**J**) Sperm count.

We examined the effect of *Utf1* deletion on spermatogenesis in 8-week-old males. The body weight was similar between different genotypes (Fig. 5A). Testis weight was significantly reduced in both heterozygous and cKO animals compared to wild type (Fig. 5B). Sperm count of cKO males was decreased by approximately 40% compared to wild type, but sperm count of *Utf1* heterozygous males was not significantly reduced (Fig. 5C). Histological analysis revealed that approximately 20% of seminiferous tubules from 8-week-old cKO males exhibited severe defects in spermatogenesis, while the remaining tubules appeared to be normal (Fig. 5D). Seminiferous tubules from *Utf1* ^+/−^ testes contained a full spectrum of spermatogenic cells and lacked apparent histological defects (Fig. 5E). However, seminiferous tubules from *Utf1* cKO testes exhibited a variety of defects. Some mutant tubules displayed a general paucity of germ cells (the middle tubule in Fig. 5F) and other tubules (indicated by asterisks) lacked almost all germ cells (Fig. 5F). Some mutant tubules showed a localized loss of all germ cells, including spermatogonia, spermatocytes, and round spermatids (Fig. 5G). In the remaining mutant tubules, loss of an entire middle layer of germ cells - spermatocytes - was observed (Fig. 5H). These testicular defects are heterogeneous and suggest that UTF1 plays an important role in spermatogenesis.

Mating tests showed that *Utf1* cKO males exhibited significantly reduced fertility (Table 2). Wild type males sired seven pups per litter on average, in contrast, *Utf1* cKO males only produced two offspring per litter on average. The dramatic reduction in the fertility of *Utf1* cKO males is consistent with the reduced sperm count.

**Table 2:** Reduced fertility in *Utf1* cKO males^a^.

Genotype	No. of litters	No. of pups	litter size^b^
WT (n = 3 males)	8	55	6.9 ± 1.1
*Utf1* cKO (n = 3 males)	12	23	1.9 ± 1.2^c^

^a^Each male was housed with two females at the age of 8-weeks for 2–3 months. Pups born were checked and recorded daily.

^b^Litter size is shown as average ± s.d.

^c^The p vale is 0.0001 by Student’s *t*-Test.

Given that not all tubules in relatively young adult cKO mice exhibited abnormalities, we evaluated whether spermatogenic defects would become more severe with age. The testes from 6-month-old cKO testes remained smaller than wild type on average (Fig. 5I), although the relative difference was reduced compared to the 8-week-old group. The sperm count of cKO animals was indistinguishable from that of wild-type males (Fig. 5J). Histological analysis of testes revealed that tubules with abnormal morphology were rare ( < 1%) in both wild-type or cKO 6-month-old adult males. These results suggest that spermatogenesis in *Utf1* cKO testes may recover over time due to the activity of spermatogonial stem cells in the intact regions of the seminiferous epithelium.

## Discussion

Here we report that UTF1 plays important roles during two stages of mouse male germ cell development: embryonic gonocyte development and postnatal spermatogenesis. Inactivation of *Utf1* results in a significant reduction in the number of gonocytes at birth. Two potential mechanisms may explain the observed gonocyte depletion in *Utf1* cKO mice: reduction of the founder population of PGCs or a defect in PGC proliferation. Because *Prdm1* is required for PGC specification, *Prdm1*-Cre-triggered deletion of *Utf1* is expected to occur concomitantly with PGC specification. Therefore, the reduction in gonocytes could result from a defect in the specification of PGCs from epiblast cells, leading to a smaller founding population of PGCs. *Utf1* cKO female mice are fertile and display no apparent histological defects in ovaries (data not shown). A mild reduction in the number of PGCs prior to sex determination might not have an observable effect on oogenesis, as fetal oocytes are present in excess, and two thirds of fetal oocytes become eliminated before birth^24^. On the other hand, the fertility of *Utf1* cKO female mice argues against an exclusive role for UTF1 in PGC specification. Alternatively, loss of UTF1 may affect PGC proliferation. PGCs proliferate during migration to the genital ridges and are enclosed in seminiferous cords in males to become gonocytes, which continue to proliferate and enter mitotic arrest at E16 in males^3^. Studies in ES cells and iPS cells have revealed a role for *Utf1* in cell proliferation^10,25^. Therefore, paucity of gonocytes in *Utf1* cKO testes may result from reduced proliferation by mitosis prior to late-gestational mitotic arrest of male germ cells.

Adult *Utf1* cKO males exhibit defects in spermatogenesis in a subset of seminiferous tubules. Shortly after birth, mouse gonocytes resume mitotic divisions and differentiate, with some becoming spermatogonial stem cells and the remainder developing into spermatocytes^3^. The paucity of germ cells (Fig. 5F) or localized loss of all stages of germ cells (Fig. 5G) in *Utf1* mutant tubules could be due to the reduction or loss of gonocytes at birth. As a more likely scenario, loss of spermatocytes and the presence of elongated spermatids from the previous round of spermatogenesis in the same tubules suggest that gonocytes might fail to produce spermatogonial stem cells capable of differentiation (Fig. 5H). Recovery of spermatogenesis in older *Utf1* cKO mice suggests that unaffected spermatogonial stem cells have the capacity to repopulate adjacent germ cell-depleted regions of seminiferous tubules. We tried to distinguish these two possibilities (defects in proliferation of gonocytes vs spermatogonial stem cells) by using the *Ddx4*-Cre, which begins to express at E15^26^. However, breeding of *Ddx4*-Cre male and *Utf1* ^fl/fl^ female mice failed to produce the desired *Utf1* ^fl/+^ *Ddx4*-Cre offspring but instead resulted in *Utf1* ^+/−^ *Ddx4*-Cre (genotype of tail genomic DNA) offspring, demonstrating global deletion of the *Utf1* floxed allele. The global deletion of the *Utf1* ^fl^ allele was likely due to the close proximity of the two loxP sites, the increased chromatin access of the *Utf1* locus, and leaky *Ddx4*-Cre expression in somatic tissues.

The phenotype of *Utf1*-deficient mice depends on the genetic background. On a complete C57BL/6J background, *Utf1*-deficient mice are significantly smaller than wild-type littermates and all die within two days after birth^13^. *Utf1* is expressed in extraembryonic ectodermal cells and its deletion causes a developmental delay of the placenta^5,13^. Therefore, the neonatal lethality phenotype most likely attributes to placental insufficiency. However, on a hybrid genetic background (75% ICR and 25% C57BL/6J), *Utf1*-deficient mice are viable and fertile albeit still smaller than wild-type mice^13^. In this study, we find that, on a mixed background (FVB/N, C57BL/6, and 129), inactivation of *Utf1* causes a much more severe phenotype - embryonic lethality by E10.5. Genetic backgrounds can potentially influence the phenotypic expression of specific genes^27,28^. Indeed, the phenotypes of an increasing number of mouse mutants are strongly influenced by genetic background^29–32^. It is known that testicular germ cell tumors arise preferentially on the 129 strain background^18^. DND1 was also identified as a chromatin-associated protein in our proteomics screen (Table 1) and its mutation is responsible for the formation of testicular germ cell tumors on the 129 background^18^. Intriguingly, the role of DND1 in the regulation of mitotic arrest in male embryonic germ cells is strongly modulated by the genetic background^17^. The variable phenotypes of *Utf1*-deficient mutants on different genetic backgrounds is therefore likely due to the presence of different alleles of genes involved in the UTF1-mediated transcriptional and epigenetic pathways.

## Methods

### Ethics statement

All experimental protocols were approved by the Institutional Animal Care and Use Committee (IACUC) of the University of Pennsylvania. All the methods were carried out in accordance with the approved guidelines.

### Chromatin purification and proteomic screen

Chromatin was isolated from 100 mg of E14.5-E16.5 testes or ovaries as described previously^14^. 75 μg of chromatin-associated proteins from testes or ovaries were separated on 10% SDS-PAGE gels and stained with Coomassie blue. Each gel lane was cut into 10 slices, digested with trypsin and subjected to HPLC followed by MS/MS at the University of Pennsylvania Proteomic Core Facility. All spectra from 10 MS/MS runs per sample were pooled and analyzed using Scaffold 4 software (Proteome Software, Portland, Oregon), producing E14.5-E16.5 testis and E14.5-E16.5 ovary datasets. Mass spec data for chromatin-associated proteins from P6 testes were generated previously^14^.

### Generation of *Utf1*^fl^ mice

For *Utf1* targeting construct generation, three *Utf1* genomic DNA fragments were amplified from a BAC clone (RP23-182C11) template by high-fidelity PCR (Fig. 3A). A floxed neomycin-resistance cassette was subcloned 5′ of the *Utf1* gene, and a *loxP* site was added between exons 1 and 2 by PCR. After validation of the gene targeting plasmid (pUtf1Ex1Flox) by sequencing, the *Kpn*I-linearized construct was used to electroporate V6.5 (C57BL/6 × 129S4/SvJae) mouse ES cells^33^. ES cells were cultured in the presence of G418 (for positive selection) and ganciclovir (for negative selection). Among 96 G418-resistant ES clones, 18 had undergone homologous recombination at both homology arms. Two *Utf1* ^*fl/+*^ ES clones (Utf1B9 and Utf1C12) were injected into blastocysts, which were transferred to pseudopregnant female mice. Matings of male chimeras with C57BL/6J females confirmed germline transmission of the *Utf1* ^*fl*^ allele. Genotyping for the floxed allele (181 bp) was performed by PCR with primers 5′-GTGGAGCAAGGTAGGAGCA-3′ and 5′-CAGGTTCGTCATTTTCCGCA-3′. Following Cre-mediated recombination, the *Utf1* knockout allele (465 bp) was assayed by PCR with primers 5′-CAGAGTGTCGGTGCTCGTAA-3′ and 5′-GTTCAAGCCCCTAGTCACAAATC-3′. *Prdm1-Cre* and *Actb-Cre* transgenic mice were purchased from Jackson Laboratory (Stock numbers: 008827 and 003376, respectively)^22,23^. The *Prdm1-Cre* strain had been backcrossed to C57BL/6 four times by the donating investigator, and *Actb*-Cre transgenic mice were on a FVB/N background.

### UTF1 antibody production

The cDNA sequence encoding the N-terminal 200 amino acids of mouse UTF1 was subcloned into the pQE-30 expression construct (Qiagen, Valencia, CA), and the resulting 6xHis-UTF1 fusion protein was expressed in M15 bacteria carrying the pREP4 lac repressor plasmid and purified using Ni-NTA agarose. Two rabbits were inoculated with the recombinant protein, resulting in antisera UP2390 and UP2391 (Cocalico Biologicals, Reamstown, PA). Antisera were affinity-purified against the UTF1 fusion protein prior to use. Other antibodies used for western blotting: Anti-histone H3 monoclonal antibody (1:10,000, Cat No. 05-499, clone 6.6.2, Millipore), ACTB monoclonal antibody (1:50,000, Cat No. A5441, clone AC-15, Sigma), and goat anti-LIN28A (1:3,000, Cat No. AF3757, R&D systems). Immuno-detection was performed using horseradish peroxidase-conjugated secondary antibodies and ECL Select detection reagents (Amersham).

### Histology and immunostaining

For histological analysis, testes were fixed in Bouin’s fixative, dehydrated, and embedded in paraffin. 8-micron sections were collected and stained with hematoxylin and eosin before mounting with Permount (Fisher Scientific, Pittsburgh, PA). Slides were imaged on a Leica DM5500B microscope with a DFC450 camera (Leica Microsystems, Buffalo Grove, IL).

For immunofluorescence, testes were fixed in 4% paraformaldehyde at 4 °C for 3–4 hours and cut into 8-micron sections in a cryostat. Slides were blocked with 10% goat serum in Tris buffered saline containing 0.1% Tween-20. The slides were incubated with a 1:200 dilution of the purified anti-UTF1 antibody at 37 °C overnight followed by one-hour incubation with a 1:200 dilution of FITC-conjugated goat anti-rabbit IgG secondary antibody. Slides were mounted in VectaShield with DAPI (Vector Laboratories, Burlingame, CA) and imaged with an ORCA-Flash4.0 digital camera (Hamamatsu Photonics, Hamamatsu City, Japan) on a Leica DM5500B microscope.

### Gonocyte quantification and sperm count

Testes from individual animals were cut into 8-micron sections and placed on slides in groups of 5 sections with each approximately 80 microns apart. After staining with DAPI, each section was imaged, and the images stitched to create full mosaics of each section. Gonocytes were counted in all seminiferous tubule cross-sections. Per genotype, testis sections from several animals were quantified. Student’s *t*-test was used to calculate *p*-values. Sperm count of cauda epididymides was performed as previously described^34^.

### Statistical methods

For each genotype, at least three animals were analyzed. All variables were presented as mean ± SD. P-values were calculated by Student’s *t*-Test and *p* values of less than 0.05 were considered statistically significant.

## Electronic supplementary material

Supplementary Figure S1
