# Supplementary material for: Embryonic lethality and defective male germ cell development in mice lacking UTF1
Source: Sci Rep. 2017 Dec 8;7:17259. doi: 10.1038/s41598-017-17482-z (PMC5722945; doi:10.1038/s41598-017-17482-z)
Supplement: Supplementary file 1 — Supplementary Figure S1 [file 41598_2017_17482_MOESM1_ESM.pdf]

Supplementary Information

Manuscript title: Embryonic lethality and defective male germ cell development in mice lacking UTF1

Seth D. Kasowitz, Mengcheng Luo, Jun Ma, N. Adrian Leu & P. Jeremy Wang

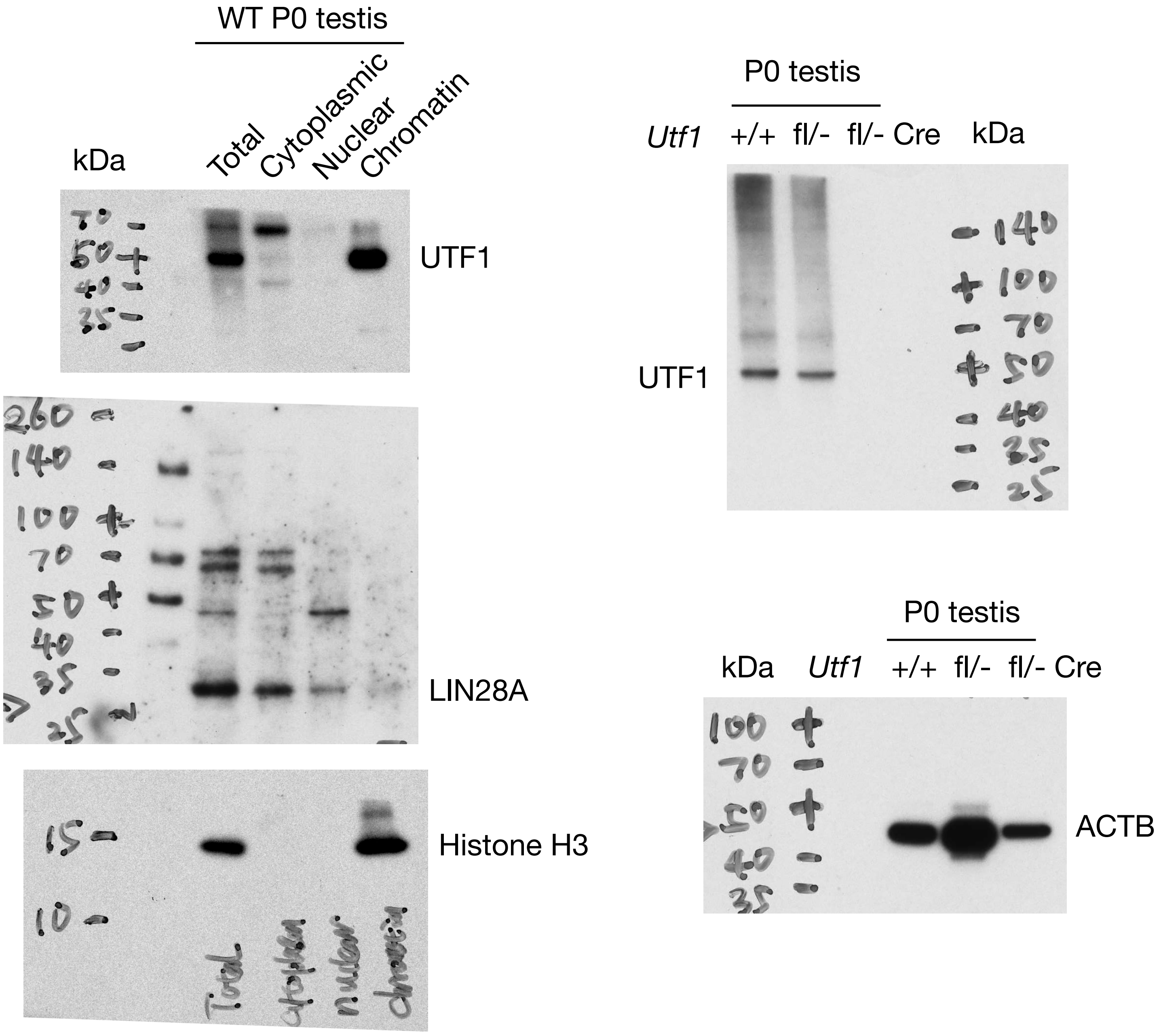

Supplementary Figure S1
Full-length Western blots for cropped blots shown in
Figure 2A (left) and Figure 3D (right)
